# On Quantum Superstatistics and the Critical Behavior of Nonextensive Ideal Bose Gases

**DOI:** 10.3390/e20100773

**Published:** 2018-10-09

**Authors:** Octavio Obregón, José Luis López, Marco Ortega-Cruz

**Affiliations:** Departamento de Física, División de Ciencias e Ingenierías Campus León, Universidad de Guanajuato, A.P. E-143, C.P. 37150 León, Guanajuato, Mexico

**Keywords:** superstatistics, Bose–Einstein condensation, Non-Additive Entropies

## Abstract

We explore some important consequences of the quantum ideal Bose gas, the properties of which are described by a non-extensive entropy. We consider in particular two entropies that depend only on the probability. These entropies are defined in the framework of superstatistics, and in this context, such entropies arise when a system is exposed to non-equilibrium conditions, whose general effects can be described by a generalized Boltzmann factor and correspondingly by a generalized probability distribution defining a different statistics. We generalize the usual statistics to their quantum counterparts, and we will focus on the properties of the corresponding generalized quantum ideal Bose gas. The most important consequence of the generalized Bose gas is that the critical temperature predicted for the condensation changes in comparison with the usual quantum Bose gas. Conceptual differences arise when comparing our results with the ones previously reported regarding the *q*-generalized Bose–Einstein condensation. As the entropies analyzed here only depend on the probability, our results cannot be adjusted by any parameter. Even though these results are close to those of non-extensive statistical mechanics for q∼1, they differ and cannot be matched for any *q*.

## 1. Introduction

Entropy is one of the most useful concepts in physics. Its meaning and interpretation in the realm of statistical mechanics has led to a beautiful understanding of the microscopic properties of thermodynamic systems, and the mathematical properties of entropy also allow defining an arrow of time, namely the direction in which physical processes occur. Future means increasing entropy. Despite the successful use of entropy in the usual form that we know, namely the Boltzmann–Gibbs one, several modifications have also been considered [1,2,3,4,5,6,7]. Such considerations were inspired by the observation of physical systems, which do not accept the usual modeling with the standard form of entropy or, in other words, the usual probability distributions of states (see for instance [8] and the references therein). One characteristic of some generalized entropy measures is the dependence of one or more parameters that can be adjusted depending on the physical system [9]. With such unusual physical systems in mind, we consider in this work non-equilibrium systems characterized by spatiotemporal fluctuations on an intensive quantity, usually the temperature [10]. Superstatistics considers these fluctuations and takes them into account when estimating the probability of the occurrence of a state in a particular configuration. The starting point is the derivation of entropy from a generalized Boltzmann factor [11,12,13,14], and the probability distribution can be deduced by maximizing the corresponding entropy; the particular case of some generalized quantum distributions can be found in [15,16,17].

We will deal with the quantum statistics of two entropies that depend only on the probability [12,13,14]. Some interesting implications of this particular generalized entropy have been studied in [12,18,19,20,21]. We will consider particularly the thermodynamic properties of quantum ideal Bose gases. Those thermodynamic properties can be calculated if the probability density of states of the determined energy is known, a probability which can be derived from entropy. We will follow this path in order to estimate the critical temperature where the Bose condensation occurs for a quantum system characterized by the generalized statistics depending only on the probability [12,14]. This analysis has already been considered for the quantum statistics of *q*-generalized entropies, and quite interesting results arise [22,23,24,25,26,27,28,29,30,31,32,33,34,35,36,37,38,39]. This particular result will also be explored here for other generalized entropies. The entropies we consider here, instead, depend only on the probability and do not have any adjusted parameter [12,18]. These entropies are derived through a generalized Boltzmann factor that takes into account small thermal fluctuations, and consequently, the form of the entropy depends on the assumed thermal distribution [10]. This fact allows identifying the nature of the differences in the thermodynamical properties of the usual (extensive) and the nonextensive quantum systems analyzed here. Even when our results are close to those predicted by the *q*-entropy for q∼1, they do not coincide with these previous predictions.

The general structure of this work is as follows. First, in Section 2, we briefly review the superstatistics framework where we define generalized Boltzmann factors from which generalized entropies that depend only on the probability can be derived. In Section 3, we first review the thermodynamic properties that define the Bose–Einstein condensation for the usual quantum statistics and for the quantum ideal gas, in particular the consequences of the quantum statistics in the occupation number, namely the number of particles in determined energy states; afterwards, we explore the thermodynamic consequences of the same ideal gas, but influenced by the modified statistics. In Section 4, we present a discussion and conclusions of the main results in our work.

## 2. Generalized Entropies

We have already mentioned that there exist several non-extensive generalizations to entropy; we have remarkable examples in [1,2,3,4,5,6]. We will deal with a special class of generalized entropies, depending only on $p_{l}$, arising in the realm of superstatistics [10,12], those inspired by non-equilibrium processes, like systems with spatiotemporal fluctuations, not far from equilibrium, in some intensive quantity, which we will choose to be the temperature. Considering a distribution of temperature f(β), a generalized Boltzmann factor B(E) can be calculated, which takes into account these fluctuations as follows:

(1) $$B\left(E\right)=\int_{0}^{\infty }f\left(\beta \right)e^{-\beta E}d\beta ,$$

where *E* is the corresponding energy, and when $f\left(\beta \right)=\delta \left(\beta -\beta_{0}\right)$, we get the usual Boltzmann factor. The procedure of obtaining Boltzmann factors from different distributions can be reviewed in [10,18], and here, we show one particular example. Let us consider a Gamma distribution depending on the parameter $p_{l}$, which will be further identified with the probability,

(2) $$f_{p_{l}}\left(\beta \right)=\frac{1}{\beta_{0}p_{l}\Gamma \left(1/p_{l}\right)}{\left(\frac{\beta }{\beta_{0}}\frac{1}{p_{l}}\right)}^{\frac{1-p_{l}}{p_{l}}}\exp\left[-\frac{\beta }{\beta_{0}p_{l}}\right],$$

where $\beta_{0}$ is the average inverse temperature. Integrating this distribution, we get its corresponding Boltzmann factor (1):

(3) $$B_{p_{l}}\left(E\right)={\left(1+p_{l}\beta_{0}E\right)}^{-\frac{1}{p_{l}}}\;.$$

It was shown in [10,18] that for several distributions, after expanding for small $p_{l}\beta_{0}E$, the generalized Boltzmann factor leads to the same first correction term; such an expansion for the case of our distribution here is:

(4) $$B_{p_{l}}\left(E\right)=e^{-\beta_{0}E}\left(1+\frac{1}{2}p_{l}E^{2}\beta_{0}^{2}-\frac{1}{3}p_{l}^{2}\beta_{0}^{3}E^{3}+,\;\cdots \right),$$

and the entropy corresponding to (3) is given by:

(5) $$S_{1}=k\sum_{l=1}^{\Omega }\left(1-p_{l}^{p_{l}}\right)\;,$$

where Ω is the total number of microstates and $p_{l}$ will be later identified with the probability; thus, we will have the constraint $\sum_{l=1}^{\Omega }p_{l}=1$. We have labeled this entropy with the subindex 1, as we will consider in this work another entropy following from another distribution f(β), in which basically $p_{l}$ is changed by $-p_{l}$ in (2), and consequently, a different Boltzmann factor and a different entropy arise [12,18]; such entropy is given by:

(6) $$S_{2}=k\sum_{l=1}^{\Omega }\left(p_{l}^{-p_{l}}-1\right)\;.$$

These entropies can be expanded, and both are equal to the Boltzmann–Gibbs entropy (Shannon) at first order. We have for instance that (5) can be written as:

(7) $$-\frac{S}{k}=\sum_{l=1}^{\Omega }\left[p_{l}\ln p_{l}+\frac{{\left(p_{l}\ln p_{l}\right)}^{2}}{2!}+,\;\cdots \right]\;.$$

The probability distribution can be obtained by maximizing the following functional:

(8) $$\Phi =\frac{S}{k}-\gamma \sum_{l=1}^{\Omega }p_{l}-\beta \sum_{l=1}^{\Omega }p_{l}^{p_{l}+1}E_{l}\;,$$

where γ and β are Lagrange multipliers related to constraints in probability and energy. We have consequently that the function defining the generalized probability $p_{l}\left(\beta E_{l}\right)$ is given implicitly by:

(9) $$1+\ln p_{l}+\beta E_{l}\left(1+p_{l}+p_{l}\ln p_{l}\right)=p_{l}^{-p_{l}}\;.$$

It is not possible to express analytically the probability as a function of energy, namely $p_{l}\left(\beta E_{l}\right)$, but we can, and will, use an approximation given by the best fitting adjustment of the inverse function of energy in terms of the probability; we will call this probability distribution $p_{l}=g\left(\beta E_{l}\right)$, where:

(10) $$g\left(\beta E_{l}\right)=e^{-\beta E_{l}}\left(a+bx+cx^{2}+dx^{3}+ex^{4}\right)\;,$$

and the constants, a,b,c,d,e, are the fitting parameters, whose specific values are shown in Appendix A. As we will use two entropies related to two different distributions, each one of them will have their corresponding fitting parameters; we name $p_{l_{I}}=g_{I}\left(\beta E_{l}\right)$ the probability related to entropy (5) and $p_{l_{II}}=g_{II}\left(\beta E_{l}\right)$ the probability distribution corresponding to entropy (6). In the next section, we will generalize the probability distributions to their quantum counterparts and particularly explore the properties of systems obeying the generalized Bose–Einstein statistics. For completeness, we will exhibit also the corresponding generalized Fermi–Dirac distribution. The differences between the probability distributions that define our entropies (10) and the ones defined by the nonextensive Tsallis *q*-statistics have been shown in [14]. For values not so far from q=1, above and below, our distributions behave similarly to those of *q*-statistics, but they are not exactly equal for any value of *q*. They are conceptually different.

## 3. Ideal Extensive and Non-Extensive Quantum Bose Gases

In this section, we will firstly review some thermodynamic properties of quantum ideal Bose systems obeying the usual quantum statistics, and afterwards, we introduce the generalized quantum statistics and show that the generalized statistics brings new important thermodynamic consequences.

### 3.1. Usual Quantum Statistics

Let us briefly review the case of the usual quantum ideal Bose gas [40,41]. The number of particles is:

(11) $$N=\sum_{\epsilon }\frac{1}{z^{-1}e^{\beta \epsilon }-1}\;,\;z=e^{\beta \mu }\;,$$

where the statistics of the mean occupation number is given by the expression:

(12) $$n_{\epsilon }=\frac{1}{z^{-1}e^{\beta \epsilon }\pm 1}\;,$$

where the sign in the second term in the denominator corresponds to Bose–Einstein (BE) statistics for −1 and Fermi–Dirac (FD) statistics for +1. Before changing the sum in (11) into an integral, care should be taken because we should not give a zero statistical weight to the state with (ϵ=0); therefore, the first term in the sum is extracted, and we will have in particular for the BE statistics:

(13) $$\frac{N}{V}=\frac{2\pi }{h^{3}}{\left(2m\right)}^{3/2}\int_{0}^{\infty }\frac{\epsilon^{1/2}d\epsilon }{z^{-1}e^{\beta \epsilon }-1}+\frac{1}{V}\frac{z}{1-z}\;,$$

where the density of states in the energy space a(ϵ) is deduced from the fact that the particles do not feel interactions among them and:

(14) $$a\left(\epsilon \right)d\epsilon =\frac{2\pi V}{h^{3}}{\left(2m\right)}^{3/2}d\epsilon \;.$$

A change of variable is made x=βϵ, and we get:

(15) $$\frac{N}{V}=\frac{1}{\lambda^{3}}g_{3/2}\left(z\right)+\frac{1}{V}\frac{z}{1-z}\;,$$

where $\lambda =\frac{h}{{\left(2\pi mkT\right)}^{1/2}}$ and $g_{\nu }\left(z\right)$ is the well-known Einstein function. If an expansion of the statistical factor in powers of $\;ze^{-x}$ is performed, we get:

(16) $$\frac{N}{V}=\frac{1}{\lambda^{3}}\left[z+\frac{z^{2}}{2^{3/2}}+\frac{z^{3}}{3^{3/2}}+\frac{z^{4}}{4^{3/2}}+\cdots \right]+\frac{1}{V}\frac{z}{1-z}\;.$$

At this point, we need to fix the limiting value of *z*. For z<<1, the expansion behaves like *z*, and this equation is used to determine *z* itself. When ν>1, the function $g_{\nu }\left(z\right)$ converges, and as z→1, it approaches the Riemann zeta function ζ(ν). The function $g_{\nu }\left(z\right)$ grows monotonically, so the maximum value of $g_{\nu }\left(z\right)$ is precisely ζ(ν). We have then the following cases: (i) when z<<1, the second term in (16), namely $\frac{N_{0}}{V}$ where $N_{0}=\frac{z}{1-z}$, is negligible and $g_{\nu }\left(z\right)$, becomes a polynomial in *z*; (ii) when z→1, the term proportional to $N_{0}$ cannot be neglected. This corresponds to the number of accumulation particles into the single state with energy ϵ=0. As the number density has an upper limiting value, when the number of particles exceeds this limit, the rest of the particles are forced to occupy also the ground state, and the condensation takes place. This limit is explicitly given by:

(17) $$N=\frac{VT_{c}^{3/2}{\left(2\pi mk\right)}^{3/2}}{h^{3}}\zeta \left(3/2\right)\;;$$

thus, the critical temperature is:

(18) $$T_{c}=\frac{h^{2}}{\left(2m\pi k\right)}{\left[\frac{N}{V\zeta (3/2)}\right]}^{2/3}\;.$$

Temperature is fixed depending on the mass of the particles in consideration. In the next section, we determine what happens with the critical temperature when the Bose ideal gas obeys the generalized statistics determined by the non-extensive entropies [12,14].

### 3.2. Generalized Quantum Statistics

**Figure 1:**
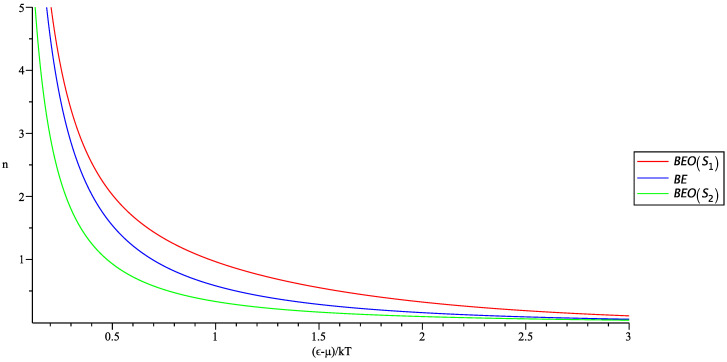
Simultaneous plot of the usual occupation number (blue line) and the generalized occupation numbers for the BEO statistics for $S_{1}$ (red line) and $S_{2}$ (green line).

**Figure 2:**
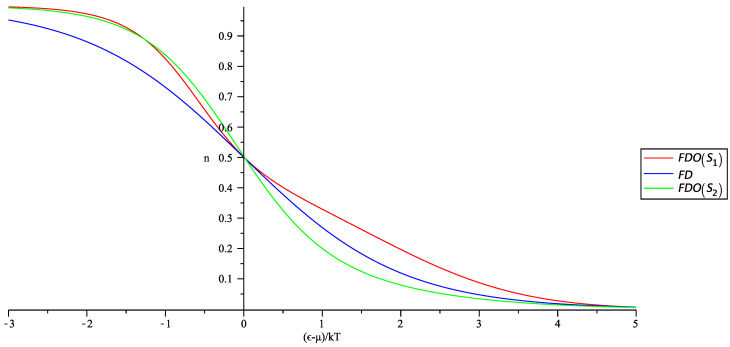
Simultaneous plot of the usual occupation number (blue line) and the generalized occupation number of the FDO statistics for $S_{1}$ (red line) and $S_{2}$ (green line).

As we have mentioned before, the probability density cannot be expressed in analytical form when maximizing its corresponding functionals, but we can approximate, in different ways, the probability distribution with an explicit function that fits the curve of the inverse function of energy in terms of probability as in Equation (9), and we can also get accordingly the generalized quantum statistics for systems that correspond to the entropies $S_{1}$ and $S_{2}$ defined above that were first proposed by O. Obregón in [12]. These generalized statistics will be called BEO and FDO for the generalized Bose–Einstein and Fermi–Dirac statistics respectively. Using the kind of approximation as in Equation (10), we find that the corresponding generalized occupation number is given by: [14]

(19) $$n_{\epsilon }=\frac{1}{e^{y}{\left[a+by+cy^{2}+dy^{3}+ey^{4}\right]}^{-1}\pm 1},\;y=\left(\beta \epsilon -\beta \mu \right)\;,$$

where −1 corresponds to BEO statistics and +1 to FDO statistics. In Figure 1 and Figure 2, we show in a single plot the occupation numbers for the usual and generalized statistics for the corresponding entropies $S_{1}$ and $S_{2}$.

We observe from Figure 1 and Figure 2 that the generalized $n_{\epsilon }$ behave slightly different from the usual; however, the BEO occupation number still will allow for condensation, and the FDO occupation number is consistent with Pauli’s exclusion principle. For completeness, we have also shown the behavior of the FDO statistics, but from now on, we will focus only on the properties of the BEO statistics. We can calculate now the density of particles assuming that the new statistics is obeyed, by rewriting the occupation number in terms of $z=e^{\beta \mu }$; it becomes:

(20) $$\frac{N}{V}=\frac{2\pi {\left(2m\right)}^{3/2}}{h^{3}}\int_{0}^{\infty }\frac{\epsilon^{1/2}d\epsilon }{a^{-1}z^{-1}e^{\beta \epsilon }{\left[1+By+Cy^{2}+Dy^{3}+Ey^{4}\right]}^{-1}-1}+\frac{N_{0}}{V}\;,$$

where y=βϵ−lnz, and we have redefined the constant fitting parameters as $B=\frac{b}{a}$, $D=\frac{d}{a}$, and so on. After the change x=βϵ, we get:

(21) $$\frac{N}{V}=\frac{2\pi {\left(2m\right)}^{3/2}}{h^{3}\beta^{3/2}}\int_{0}^{\infty }\frac{x^{1/2}dx}{a^{-1}z^{-1}e^{x}{\left[1+By+Cy^{2}+Dy^{3}+Ey^{4}\right]}^{-1}-1}+\frac{N_{0}}{V}\;.$$

In the last two expressions, we have also extracted the ϵ=0 term in the occupation number $N_{0}$, as the same situation occurs as in the usual statistics, but in this case, $N_{0}$ is given by:

(22) $$N_{0}=\frac{az[1-B\ln z+C\ln^{2}z-D\ln^{3}z+E\ln^{4}z]}{1-az[1-B\ln z+C\ln^{2}z-D\ln^{3}z+E\ln^{4}z]}\;.$$

**Figure 3:**
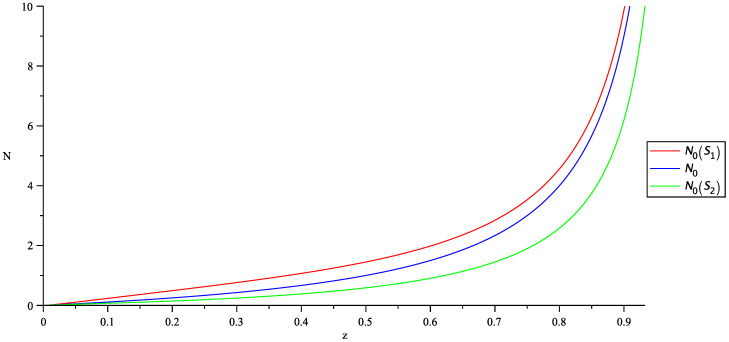
Behavior of $N_{0}\left(z\right)$ in the interval 0<z<1 for the usual probability distribution (blue line) and for the two non-extensive probability distributions corresponding to $S_{1}$ (red line $N_{0}\left(S_{1}\right)$) and $S_{2}$ (green line $N_{0}\left(S_{2}\right)$). When z<<1, $\;N_{0}\left(z\right),N_{0}\left(S_{1}\right)$ and $N_{0}\left(S_{1}\right)$ can be neglected, but when *z* is close to one, the number of particles that accumulate in the ground state grows rapidly in all cases.

In Figure 3, we plot the behavior of $N_{0}=\frac{z}{1-z}$ of the usual statistics and $N_{0}$ as in Equation (22).

Following with the calculation of the first term (integral) in Equation (21), the first term in the denominator will be rewritten by expanding the powers of y=(x−lnz) to get:

(23) $$\frac{N}{V}=\frac{2\pi {\left(2m\right)}^{3/2}}{h^{3}\beta^{3/2}}\int_{0}^{\infty }\frac{x^{1/2}\left(aze^{-x}\right)dx}{{\left[1+f_{1}+f_{2}x+f_{3}x^{2}+f_{4}x^{3}+Ex^{4}\right]}^{-1}-\left(aze^{-x}\right)}\;,$$

where the $f_{i}$ functions depend only on *z* in the following way:

(24) $$\begin{array}{c} f_{1}\left(z\right)=-B\ln z+C\ln^{2}z-D\ln^{3}z+E\ln^{4}z,\\ f_{2}\left(z\right)=B-2C\ln z+3D\ln^{2}z-4E\ln^{3}z,\\ f_{3}\left(z\right)=C-3D\ln z+6E\ln^{2}z\;,\\ f_{4}\left(z\right)=D-4E\ln z. \end{array}$$

In order to make the expression simpler, we define:

(25) $$\left[1+f_{1}+f_{2}x+f_{3}x^{2}+f_{4}x^{3}+Ex^{4}\right]=H\left(x,z\right)\;,$$

thus:

(26) $$\frac{N}{V}=\frac{2\pi {\left(2m\right)}^{3/2}}{h^{3}\beta^{3/2}}\int_{0}^{\infty }x^{1/2}\left(aze^{-x}\right)H{\left[1-\left(aze^{-x}\right)H\right]}^{-1}dx\;.$$

**Figure 4:**
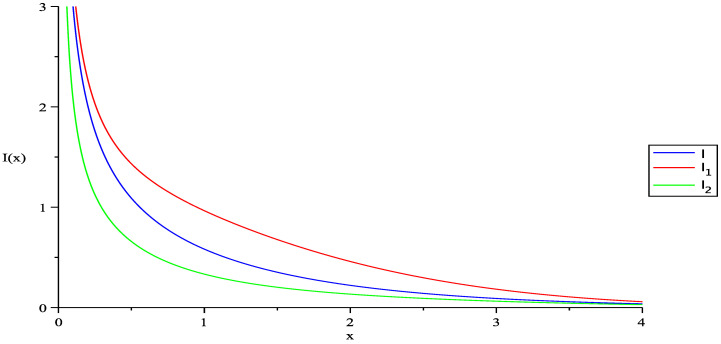
Plot of the integrands in the expressions for the usual density (blue line) and those corresponding to the densities of the modified statistics for $S_{1}$ (red line) and $S_{2}$ (green line).

After expanding the last factor when $(aze^{-x})H<1$, we have:

(27) $$\frac{N}{V}=\frac{2\pi {\left(2m\right)}^{3/2}}{h^{3}\beta^{3/2}}\int_{0}^{\infty }x^{1/2}\sum_{l=1}{\left(aze^{-x}\right)}^{l}H^{l}dx\;,$$

and using the definition of H(x,z) of Equation (25) and its powers, we can finally write:

(28) $$\frac{N}{V}=\frac{1}{\lambda^{3}}g_{3/2}\left(az\right)+\frac{1}{\lambda^{3}\Gamma \left(3/2\right)}\int_{0}^{\infty }x^{1/2}F\left(x,z\right)dx\;,$$

where F(x,z) is the remainderfunction after extracting the first contribution in terms of the Einstein function. At this point, we have succeeded in extracting the two first relevant terms in the non-extensive density. Now, it is necessary to discuss again the relevance of the density corresponding to the particles in the ground state. In order to have a better idea about the difference between the extensive and non-extensive densities, let us perform the integrals and numerically estimate their values in the upper limit as z→1; we have:

(29) $$\begin{array}{c} I=\int_{0}^{\infty }\frac{x^{1/2}dx}{z^{-1}e^{x}-1}=\frac{\sqrt{\pi }}{2}\zeta \left(3/2\right)=2.31482,\\ I_{1}=3.075,\\ I_{2}=1.463, \end{array}$$

where $I_{1}$ and $I_{2}$ are the numerical integrals using the fitting parameters for the two BEO statistics corresponding to the entropies $S_{1}$ and $S_{2}$, respectively. In Figure 4, we plot the integrands in the case of the usual statistics as in Equation (13) and for the new statistics as in Equation (21) for the two entropies. In the three cases, the density has an upper value and is bounded. We can see that $I_{2}<I<I_{1}$. As we have managed to extract the first contribution as an Einstein function, we can write:

(30) $$\begin{array}{c} \frac{N}{V}=\frac{1}{\lambda^{3}}\left[g_{3/2}\left(az\right)\pm C\right]\;, \end{array}$$

where the contribution *C* is given by:

(31) $$C=\frac{1}{\Gamma \left(3/2\right)}\int_{0}^{\infty }x^{1/2}F\left(x,z\right)dx\;,$$

and (+C) corresponds to the BEO statistics of the entropy $S_{1}$ for which the density is above the usual limiting value and (−C) corresponds to the BEO statistics of $S_{2}$, which is below the limiting value. As (az)→1, $g_{3/2}\left(az\right)\approx g_{3/2}\left(z\right)=\zeta \left(3/2\right).$ In this limit, we can estimate the critical temperatures at which the condensation will occur if the system obeys the BEO statistics of one or another generalized entropy, we will have correspondingly:

(32) $$T_{c}=\frac{h^{2}}{\left(2m\pi k\right)}{\left[\frac{N}{V\left(\zeta (3/2)\pm C\right)}\right]}^{2/3}\;,$$

and we can conclude that the critical temperature for the systems obeying the generalized statistics for one or another entropy are below the usual critical value for $S_{1}$ and above the critical value for $S_{2}$. The relation among these critical temperatures is $T_{1c}<T_{c}<T_{2c}$, where $T_{1c}$ is the critical temperature for the system obeying the generalized statistics followed by $S_{1}$ and $T_{2c}$ for $S_{2}$.

## 4. Discussion and Conclusions

We have explored some thermodynamic properties of a quantum ideal gas obeying a novel generalized statistics [12]. We considered particularly the quantum probability distribution emerging in the realm of superstatistics corresponding to the entropies of a system driven not so far from equilibrium by considering spatiotemporal thermal fluctuations. Two generalized probability quantum distributions corresponding to two different entropies that depend only on the probability were analyzed [12,13,18]. The relevant result is that the critical temperature when the condensation occurs is naturally modified if the system obeys these generalized quantum statistics. We have shown that the generalized densities (28) are also bounded in the limit z→1; therefore, the final expression for the density in this limit (30) is justified, and the critical temperature (32) follows directly. We observe that the modification of the critical temperature is a consequence of considering a different statistics and does not depend on other thermodynamic parameters as volume or particle number; this can be seen from the expression (28), which is a consequence of the mathematical form of the quantum statistical factor in the generalized occupation number (19).

Phase transitions of systems obeying modified statistics have been studied particularly for generalized *q*-statistics in [22,23,24,25,26,27,28,29,30,31,32,33,34,35,36,37,38,39], where the corresponding critical condensation temperature has also been calculated [29]. An extended study of the thermodynamic properties presented in this work and other interesting ones will be reported elsewhere.

## Appendix A. Fitting Parameters of the Generalized Probability Distributions

The fitting constant parameters in the generalized quantum probability distributions are given by:

(A1) a=1.00477,b=−0.0134648,c=0.512088,d=−0.185251,e=0.016645,

for the probability corresponding to the entropy $S_{1}$ and:

(A2) a=1.0126,b=−0.558859,c=0.270725,d=−0.048416,e=0.00302662,

for the distribution corresponding to the entropy $S_{2}$.
